# Custom-Built Vision-Assisted Arc Discharge System for Fabricating High-Q In-Line Microbubble Resonator Arrays

**DOI:** 10.3390/mi17091091

**Published:** 2026-09-16

**Authors:** Yanbin Ma, Fangjie Shu, Meng Wang, Di Yu, Yuhang Wang, Zongren Yue, Chunzhi Sun

**Affiliations:** Henan Engineering Research Center of Microcavity and Optoelectronic Intelligent Sensing, Shangqiu Normal University, No. 298 Wenhua Street, Shangqiu 476000, China; mayanbin@sqnu.edu.cn (Y.M.); wangmeng@sqnu.edu.cn (M.W.); yudi@sqnu.edu.cn (D.Y.); 19836085207@163.com (Y.W.); 16650525937@163.com (Z.Y.); sun168400@163.com (C.S.)

**Keywords:** microbubble resonator, whispering gallery mode, arc discharge, in-line array, morphological symmetry, optofluidics

## Abstract

Whispering gallery mode microbubble resonators (MBRs) are prominent optofluidic platforms, yet fabricating highly symmetric in-line arrays remains a technical challenge. Here, we demonstrate a custom-built, vision-assisted arc discharge apparatus for the deterministic fabrication of high-Q MBRs. Using finite-element simulations and microscopic observations, we reveal that asymmetric expansion is primarily driven by transverse misalignment within the plasma arc and intrinsic thermal anisotropy caused by the current crowding effect. Guided by these physical insights, we utilize real-time three-axis spatial compensation to successfully fabricate highly symmetric, cascaded in-line MBR arrays on a single continuous capillary. Optical characterization of a 16-cavity array demonstrates excellent performance reproducibility, with 12 individual microbubbles achieving ultra-high Q-factors exceeding 10^7^. This highly controllable approach provides a robust platform for distributed microfluidic sensing and integrated lab-on-a-chip applications.

## 1. Introduction

Whispering gallery mode (WGM) optical microcavities have attracted considerable attention in fundamental physics and highly sensitive detection due to their ultra-high quality (Q) factors and small mode volumes [1,2,3,4]. Among various WGM geometries, the microbubble resonator (MBR) stands out as a unique structure. By inherently integrating a hollow microfluidic channel with a highly confined optical cavity, the MBR allows for the precise sensing of gases and liquids passing through it without degrading the Q-factor [5,6]. Consequently, MBRs have demonstrated immense potential in physical sensing, biochemical analysis, and microfluidic applications [7,8,9,10]. However, the performance of MBR-based sensors heavily relies on the fabrication process, as creating highly symmetric, thin-walled microbubbles with high Q-factors remains a technical challenge.

Currently, one of the most common fabrication techniques utilizes the arc discharge from a commercial optical fiber fusion splicer to heat a silica capillary while internally pressurizing it [11,12]. Although effective, this method imposes stringent requirements on the spatial tunability of the splicer. The plasma arc generated between two electrodes does not form a strict linear heating zone; instead, it exhibits a spindle-shaped thermal field. Therefore, to achieve symmetric uniform expansion, precise three-dimensional alignment—especially along the vertical and axial directions—is mandatory to locate the exact thermal center of the arc. Unfortunately, contemporary commercial fusion splicers are strictly optimized for the automated splicing of standard optical fibers, inherently lacking the spatial degrees of freedom required for such delicate manipulations. Although several early-generation splicers equipped with flexible manual controls were successfully employed in prior works to fabricate high-quality MBRs [13,14], the prolonged discontinuation of these specific machines severely restricts the widespread adoption of this technique. S. Berneschi et al. produced microbubbles with a Q-factor of 10^7^ via a modified fusion splicer [15]. By taking out the electrodes and mounting them on a custom-built holder, they revealed that commercial fusion splicers have considerable limitations for microbubble fabrication. A precision glass processing machine can also be utilized to fabricate microbubble resonators [16]. Nevertheless, such equipment is quite costly. Furthermore, the built-in translation stages of commercial splicers offer an extremely limited axial moving range, which makes it nearly impossible to continuously fabricate a series of cascaded MBR arrays on a single capillary.

Another prevalent approach for MBR fabrication is the carbon dioxide (CO_2_) laser melting technique. In this configuration, a pressurized capillary is irradiated by a focused 10.6 μm laser beam. To ensure symmetric expansion and prevent unilateral deformation, the laser is typically split into two counter-propagating beams to heat the capillary uniformly from opposite sides [17,18,19]. While CO_2_ laser systems provide excellent control over output power and bubble morphology, they suffer from significant practical drawbacks. They necessitate the construction of complex free-space optical paths, occupying a large footprint on an optical table. Additionally, the invisible nature of the 10.6 μm wavelength makes beam alignment and focusing laborious, inherently posing safety hazards and demanding substantial experimental expertise from the operators.

To overcome the aforementioned limitations, we propose and demonstrate a custom-built, highly controllable, and cost-effective arc discharge system for MBR fabrication. Instead of relying on rigid commercial splicers or bulky free-space laser setups, our apparatus integrates a highly adjustable high-voltage arc module with a precise three-axis translation stage and a real-time microscopic imaging system. Crucially, the customized electrical circuitry permits wide-range tuning of the discharge voltage and precise modulation of the discharge duration, providing substantial degrees of freedom for tailoring the electro-thermal dynamics of the microbubble inflation. This energetic flexibility, synergistically combined with the spatial dynamic centering of the capillary, effectively eliminates the asymmetric expansion caused by thermal gradients. Using this optimized process, we stably fabricated highly symmetric MBRs exhibiting optical Q-factors exceeding 10^7^. Moreover, by breaking through the stroke limitations of traditional fusion splicers, our extended axial translation capability successfully enabled the continuous fabrication of in-line MBR arrays on a single capillary, laying a solid foundation for future multi-node distributed microfluidic sensing.

## 2. Experimental Setup

**Figure 1:**
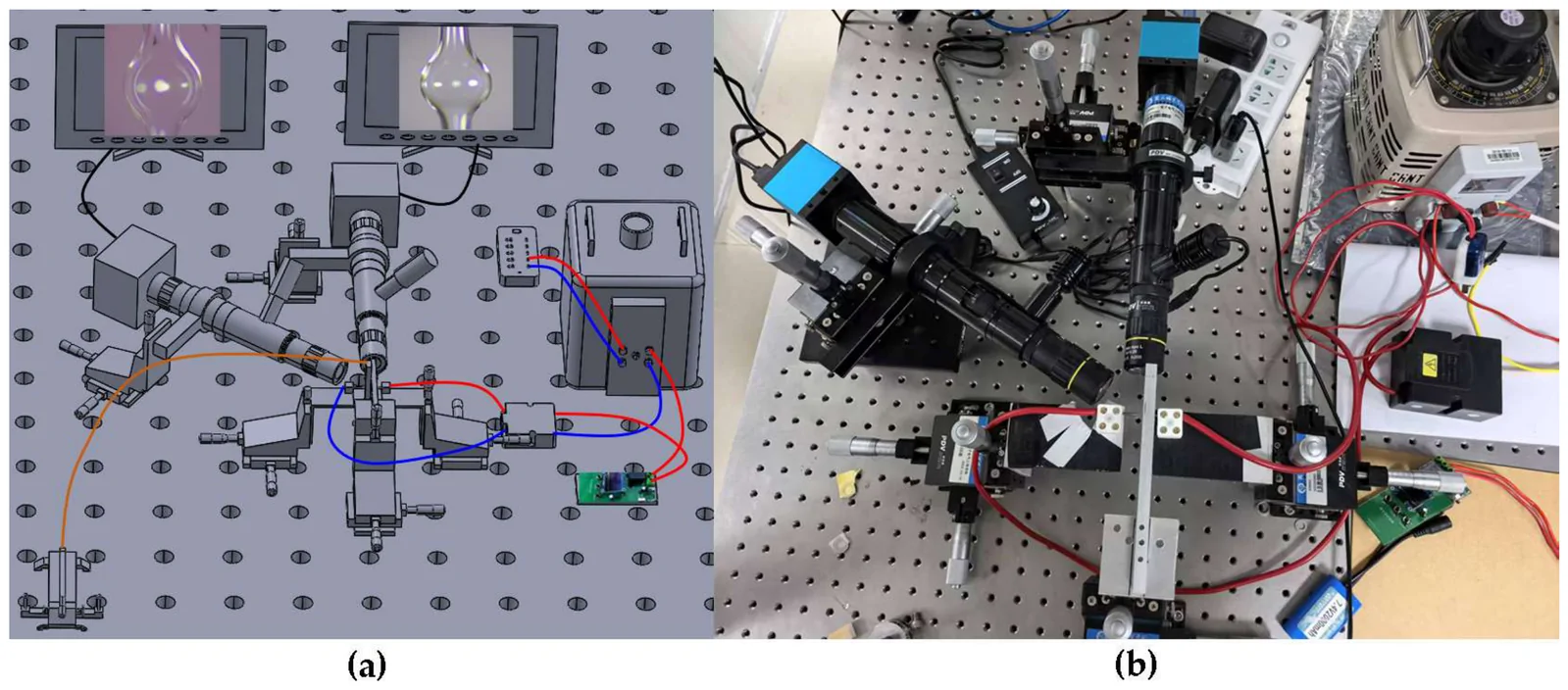
(**a**) Schematic diagram of the experimental system setup. The red and blue wires represent the live and neutral connections of the circuit, the brown wire denotes the gas path connection, and the black wire represents the display signal wire connection. (**b**) photo of the experimental setup.

We independently designed and constructed a highly tunable arc-discharge system dedicated to the fabrication of microbubble resonators (MBRs). As illustrated in Figure 1, the custom-built setup primarily consists of an AC voltage regulator, a microcontroller-based time relay, a high-voltage transformer, a pair of tungsten electrodes, a precise three-axis translation stage, a manual pneumatic pump, and a real-time microscopic imaging system.

The silica capillary is securely mounted on the manual three-axis translation stage and positioned horizontally between the two tungsten electrodes. This translation stage is a crucial component of our setup, as it provides the necessary degrees of freedom to accurately align the capillary with the optimal thermal center of the plasma arc, and allows for axial stepping to fabricate cascaded MBR arrays.

The discharge intensity and thermal energy injected into the capillary are dynamically governed by a customized electrical control module. An AC voltage regulator (0–250 V adjustable output) is connected to the primary coil of a high-voltage transformer with a turns ratio of 1:40, stepping up the mains voltage to a maximum of 8 kV to breakdown the air gap. To ensure operational safety and prevent leakage currents under high-frequency pulses, the transformer is fully encapsulated with epoxy resin and housed in a flame-retardant ABS plastic casing. In the actual fabrication process, a two-step heating strategy is employed. Initially, when the capillary wall is thick, the AC regulator output is set to 165 V to rapidly melt the silica. Once the microbubble begins to expand, the voltage is reduced to 125 V. This reduction lowers the arc intensity, enabling a gentler, highly controllable expansion and preventing catastrophic burst of the thinned wall.

Furthermore, the duration of each discharge pulse is strictly modulated by a custom-designed time-control module integrating an STC-8 microcontroller and a solid-state relay. The developed control program provides a wide adjustment range from 10 to 1000 ms with a fine resolution of 10 ms, effortlessly satisfying the requirement for millisecond-level precision in thermal control.

To provide the internal driving force for capillary expansion, a highly accessible and cost-effective manual pneumatic pump is hermetically connected to one end of the silica capillary. By manually adjusting the injected air volume, the internal relative pressure can be easily regulated and maintained at the desired level (e.g., a gauge pressure of 0.67 atm). This straightforward yet reliable pneumatic setup not only significantly simplifies the overall apparatus but also provides sufficient and stable uniform pressure to drive the inflation of the softened silica wall.

The entire melting and expansion process is monitored in situ using a monocular microscope system. The microscope features a 0.75×–5× continuous zoom host paired with a 0.5×–2× objective lens, providing a comfortable working distance of 30 to 40 mm. This large working distance not only yields clear and magnified images but also protects the lenses from thermal radiation and accidental mechanical collisions. Crucially, the microscope is equipped with a specialized industrial CCD camera featuring anti-saturation and anti-glare technologies. This ensures that the video feed does not suffer from overexposure or blackout during the intense flashes of the high-voltage arc, providing continuous and reliable visual feedback for the symmetric alignment of the microbubble.

**Figure 2:**
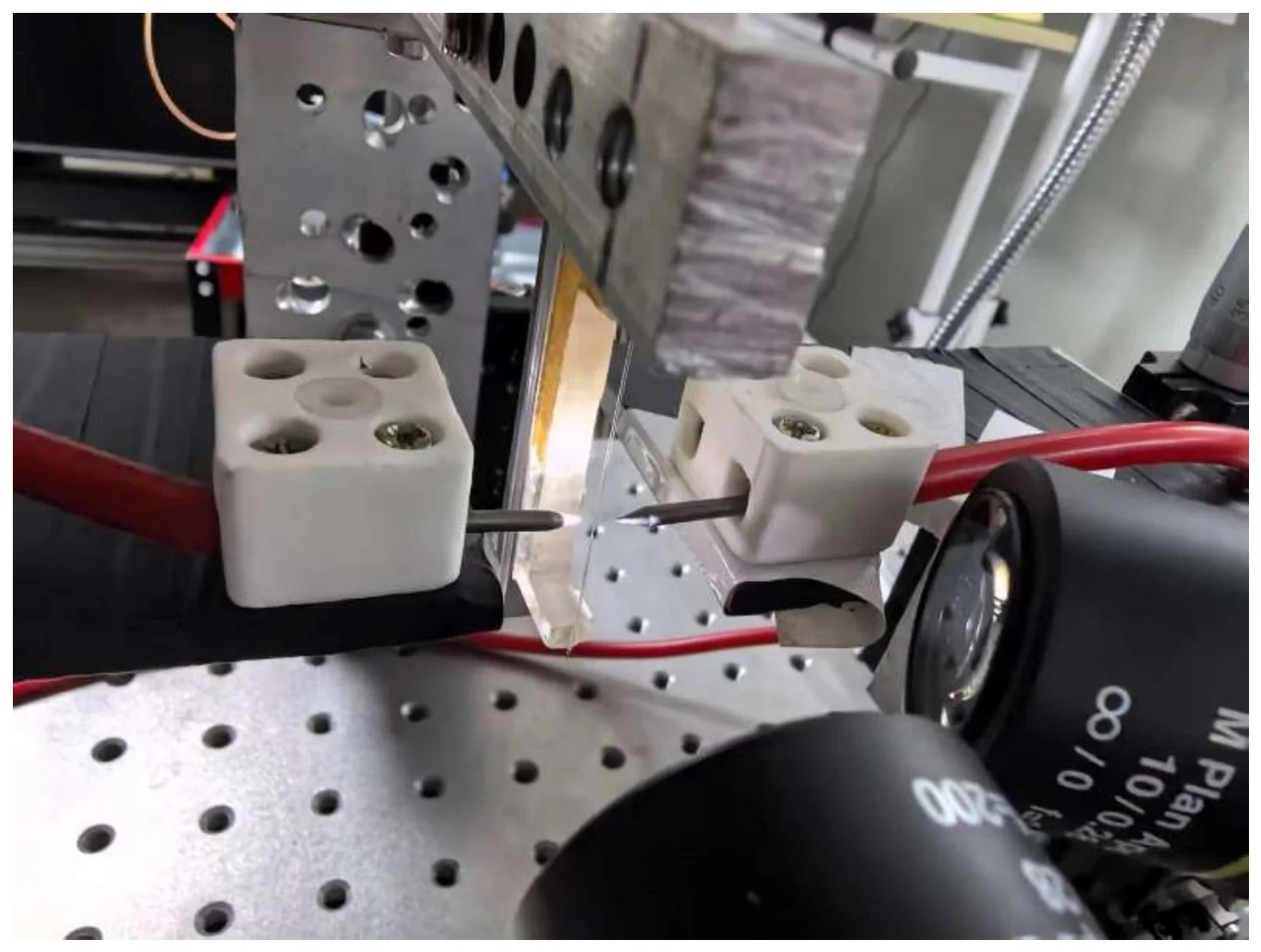
Photograph of the discharge tungsten needle area.

As depicted in the magnified view in Figure 2, the pair of tungsten electrodes is mounted on two independent three-axis translation stages, granting exceptional spatial degrees of freedom. This configuration facilitates precise tip-to-tip alignment and allows for flexible adjustment of the inter-electrode gap distance. The silica capillary is rigidly bonded to a custom-machined aluminum holder using an ultraviolet (UV) curing adhesive, protruding horizontally into the space between the two tungsten tips. Crucially, the capillary itself is secured on a separate three-axis translation stage, enabling precise spatial manipulation. Since the plasma arc generated between the electrodes does not propagate along a strict straight line connecting the two tips—but rather exhibits a curved, spindle-like spatial distribution—the ability to finely tune the capillary’s three-dimensional position is absolutely essential. This independent mobility ensures that the capillary can be dynamically aligned with the exact thermal center of the arc, which is the fundamental prerequisite for achieving symmetric bubble expansion.

To objectively evaluate the practical advantages and limitations of our custom-built apparatus, Table 1 provides a comprehensive benchmarking comparison against existing state-of-the-art MBR fabrication methods, namely commercial fusion splicers (or specialty glass processors) and CO_2_ laser heating techniques.

Commercial fusion splicers are highly automated and boast an ultra-compact footprint with fast fabrication times. However, their high equipment cost, “black-box” operational programs, and heavily restricted axial travel range make it difficult for researchers to actively intervene in the thermal symmetry control or to fabricate continuous in-line arrays. On the other hand, CO_2_ laser systems offer excellent power tunability and array capabilities, but they require significant financial investment, a large optical table footprint, and complex invisible-beam alignment. In comparison, our custom-built arc discharge system achieves a highly favorable balance. It dramatically reduces the total equipment cost to approximately 3000 USD while providing researchers with high degrees of spatial and energetic freedom. Most notably, the extended axial translation stage successfully enables the fabrication of cascaded in-line arrays with world-class Q-factors (>10^7^). It should be objectively noted that, as a manual prototype, our system requires a slightly longer fabrication time per bubble (typically 5 to 8 min) compared to automated commercial machines, and the dynamic centering relies on the operator’s manual adjustments based on visual feedback. Nevertheless, this setup provides a highly accessible, cost-effective, and reliable alternative for researchers seeking to fabricate high-performance optofluidic microcavities without the prohibitive costs of commercial equipment.

## 3. Fabrication Process

The fabrication of the high-Q microbubble resonators consists of four main phases: sample preparation, initial melting, dynamic symmetry compensation, and wall-thinning finalization.

1. Sample Preparation and Alignment:

The process begins with the thermal stripping of the polyimide coating on a standard silica capillary using the inner flame of an alcohol burner. The residual charred coating is carefully wiped away with optical lens tissue to expose a pristine silica section. For capillaries with a relatively large outer diameter exceeding 300 μm, tapering treatment can be implemented on an optical fiber tapering workstation to decrease the outer diameter to approximately 100 μm. Capillaries after tapering are conducive to the fabrication of symmetric microbubbles. The bare section is then rigidly secured onto a custom-machined aluminum mount using an ultraviolet (UV) curing adhesive. One end of the capillary is hermetically connected to the pneumatic circuit of the precision pressure pump, while the other end is sealed. Subsequently, the three-axis translation stage is manipulated to precisely position the capillary at the geometric midpoint between the two tungsten electrodes. The dual monocular microscopes are then carefully focused to provide clear, in situ observation of the target area.

2. Initial Melting and Expansion:

To provide the driving force for inflation, the internal pressure of the capillary is elevated to a gauge pressure of 0.67 atm. The AC voltage regulator is initially set to 165 V to generate a robust arc plasma. The discharge duration is precisely governed by the microcontroller, with the initial pulse width set between 150 and 200 ms. By repeatedly triggering the discharge switch, localized thermal energy is injected into the silica capillary until the softening point is reached, initiating a visible outward expansion of the capillary segment.

3. Dynamic Symmetry Compensation:

As the microbubble inflates, its morphological symmetry is continuously monitored through the side-view microscope. Due to the spindle-like spatial distribution of the arc plasma, any slight deviation of the capillary from the exact thermal center leads to an uneven thermal gradient, which inevitably causes asymmetric inflation. In practice, asymmetry occurs more frequently in the transverse direction (perpendicular to the arc axis) where the thermal boundary is narrow and steep, whereas the axial heating remains relatively uniform. Upon detecting any sign of asymmetric bulging, the capillary’s spatial position is dynamically fine-tuned via the three-axis stage to re-center it within the optimal thermal field. Subsequent discharges are then applied to actively correct and restore the structural symmetry.

4. Wall-thinning and Finalization:

As the microbubble continues to expand, its silica wall becomes progressively thinner and more susceptible to over-inflation. To prevent catastrophic rupture and ensure geometric reproducibility without relying on expensive closed-loop pressure feedback systems, a synergic step-down control strategy is implemented. First, the internal relative pressure is manually reduced from the initial ~0.67 atm to approximately 0.43 atm. Second, the single-discharge duration is decreased to a short-pulse mode of 90–120 ms.

This approach acts as a “time-gated thermal modulation”. Because the heat pulse is exceedingly brief, the silica wall only expands by a minuscule, incremental amount per discharge and instantly rigidifies once the arc is turned off. Consequently, the ultimate diameter and wall thickness are governed primarily by the precise temporal resolution of the discharge rather than strict absolute pressure stability. During these brief, high-frequency discharges, the dynamic centering process is continuously maintained. This cycle is repeated until the microbubble reaches the target dimension, successfully concluding the fabrication.

## 4. Results and Discussion

### 4.1. Thermal Field Simulation and Morphological Symmetry Analysis

**Figure 3:**
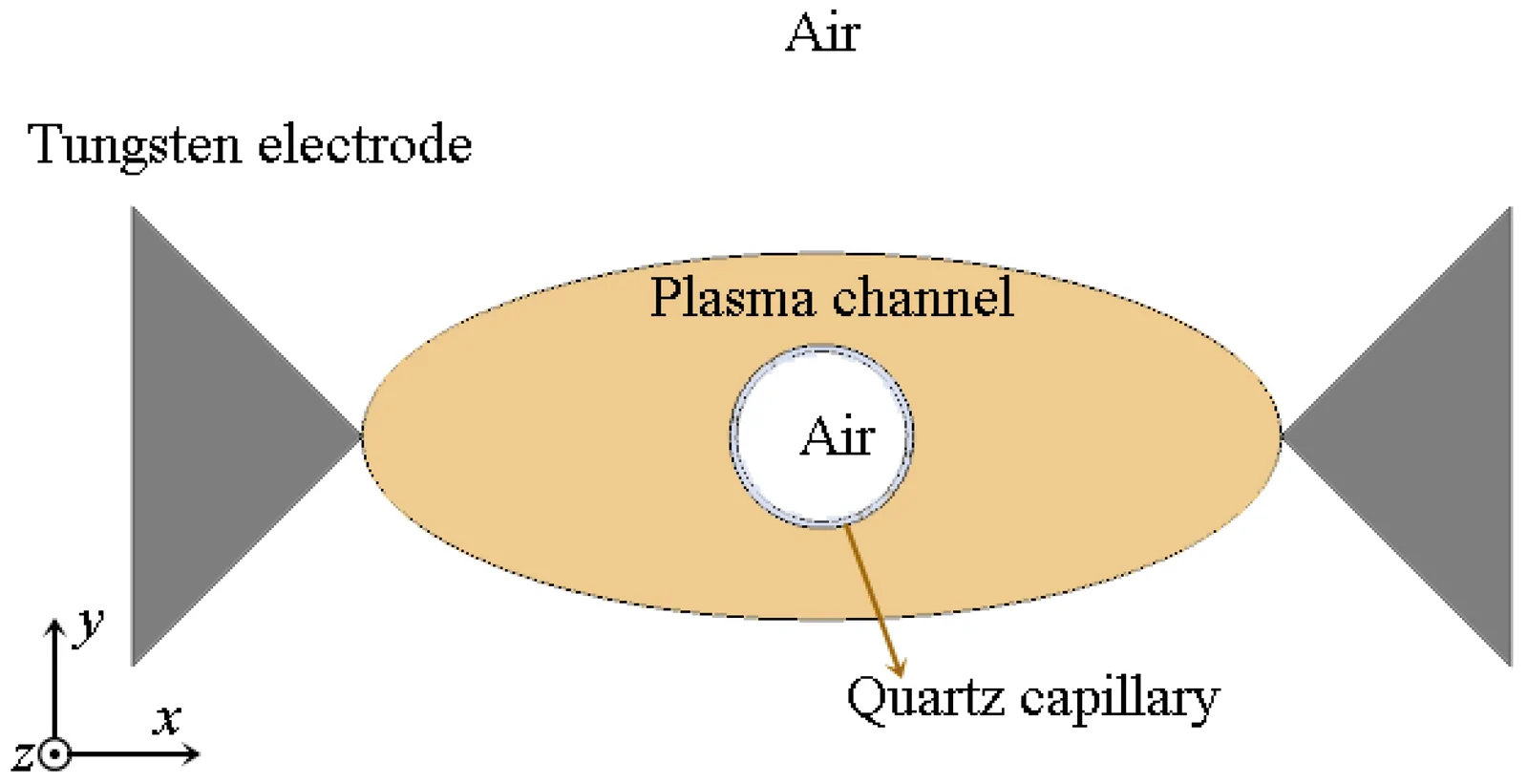
Schematic diagram of geometric structure of discharge heating model.

To theoretically investigate the spatial thermal distribution during the arc discharge and elucidate the underlying mechanisms of capillary asymmetric expansion, a two-dimensional (2D) steady-state multiphysics model was developed using finite-element method (FEM) method. The schematic diagram of the geometric structure of the model is shown in Figure 3. The numerical simulation was carried out by rigorously coupling the governing equations of electric currents and solid-fluid heat transfer. Given the extreme computational complexity and severe non-linear convergence issues associated with full magnetohydrodynamic (MHD) plasma avalanche breakdown, we adopted a widely accepted phenomenological effective heat source approach [20]. Specifically, the arc plasma was approximated as a highly conductive elliptical channel bridging the two tungsten electrodes. Rather than being arbitrarily defined, the geometric dimensions of this elliptical proxy were phenomenologically estimated based on the actual longitudinal melting zone observed on the silica capillary.

In this model, the silica capillary was assigned as a perfect electrical insulator, while the phenomenological plasma channel was defined with a high electrical conductivity to represent the ionized air. By applying a constant electric potential difference across the tungsten electrodes, the generated Joule heating density within the plasma channel was calculated and subsequently coupled as the thermal source for the heat transfer equations. This simplified yet highly effective phenomenological model circumvents the fluidic complexities of plasma while accurately capturing the essential electro-thermal coupling behavior, thereby enabling a quantitative analysis of the thermal gradients induced by both spatial misalignment and current crowding effects.

**Figure 4:**
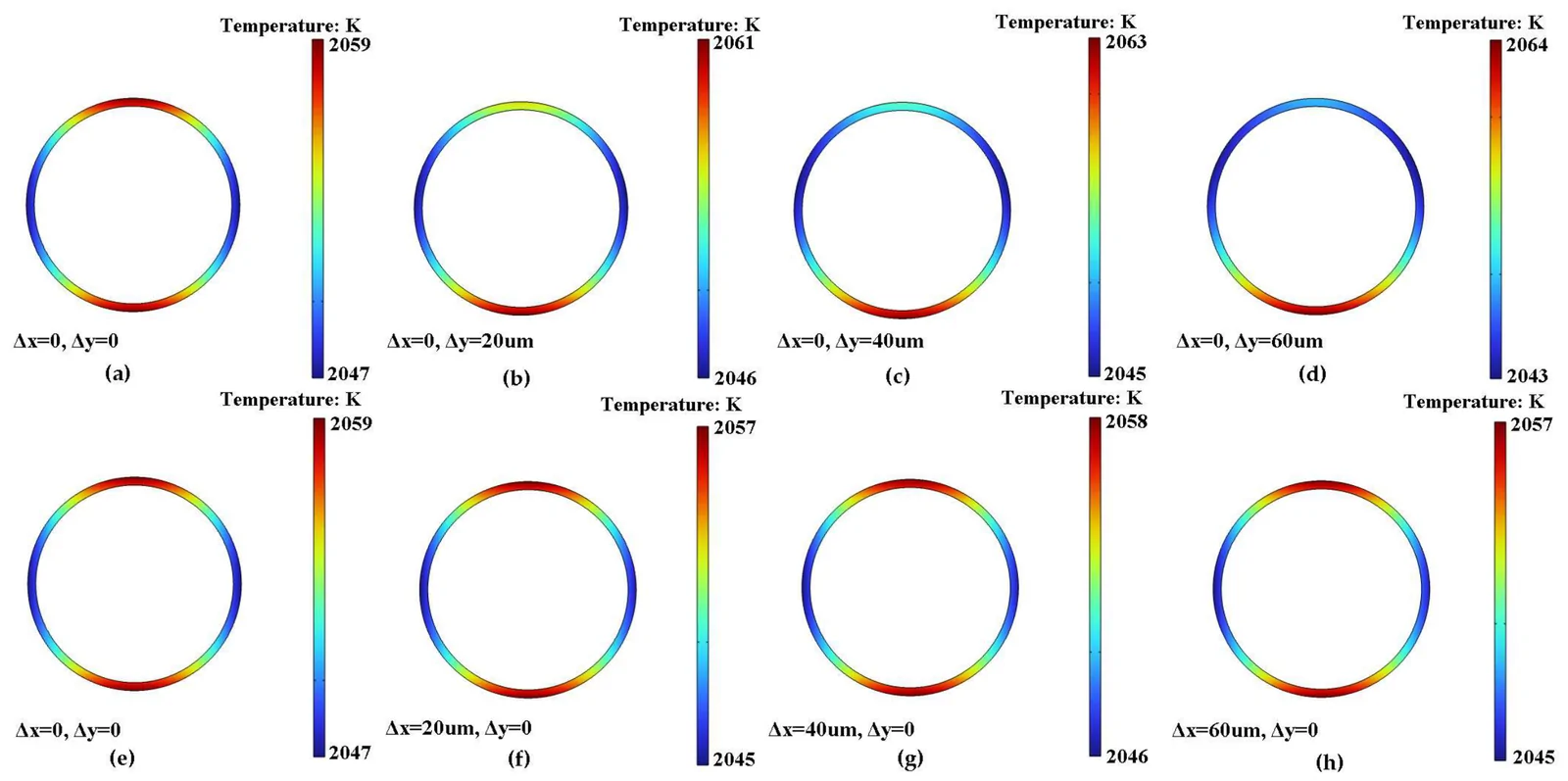
Simulated wall temperature distribution of the capillary at different positions. (**a**–**d**): Δx = 0, Δy = 20 μm, 40 μm and 60 μm; (**e**–**h**): Δx = 20 μm, 40 μm and 60 μm, Δy = 0.

Taking the midpoint of the line connecting the two tungsten electrodes as the coordinate origin (0,0), the temperature profiles along the capillary wall were simulated under the centered condition, as well as under stepped offsets of Δy = 20, 40, and 60 μm, and Δx = 20, 40, and 60 μm, respectively (Figure 4). As illustrated in Figure 4a–d, the spatial positioning of the capillary along the *y*-axis (perpendicular to the inter-electrode axis) is of paramount importance. A displacement in the y-direction severely breaks the thermal symmetry across the capillary wall, acting as the primary driver for the uneven expansion observed during fabrication. In stark contrast, Figure 4e–h demonstrate that the capillary is significantly more tolerant to offsets along the *x*-axis; even a shift of Δx = 60 μm fails to disrupt the temperature symmetry of the wall to a noticeable degree. This directional disparity in sensitivity occurs because the spindle-shaped plasma arc possesses a relatively elongated, uniform heating zone along the *x*-axis, whereas its transverse thermal gradient along the narrow *y*-axis is much steeper. These simulated findings are in perfect alignment with our empirical observations during the manual alignment process.

**Figure 5:**
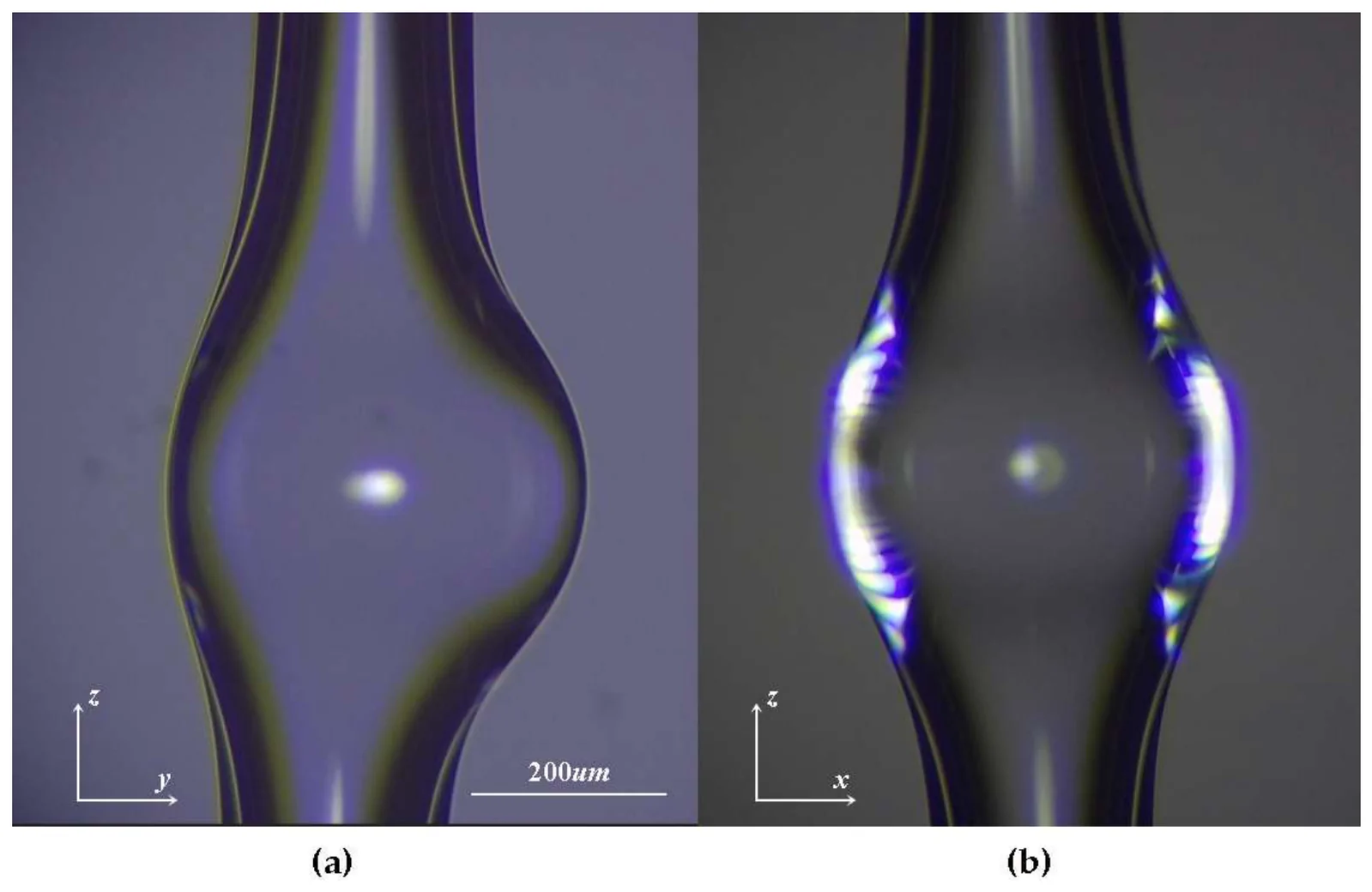
Microbubbles fabricated without precise adjustment of the capillary position during preparation. Panels (**a**,**b**) were captured with the same microscope at an identical magnification. The inconsistent backgrounds of figure (**a**) and figure (**b**) are caused by light reflection of the aluminum bracket used to fix the capillary.

During the experimental fabrication, in the absence of active fine-tuning via the translation stage, the process is highly susceptible to the unilateral deformation depicted in Figure 5. As shown, the capillary exhibits a pronounced asymmetric expansion along the *y*-axis. This defect stems directly from the spatial deviation of the capillary relative to the thermal center, which induces non-uniform thermal accumulation across the *y*-axis tube wall. Notably, this transverse asymmetry is virtually imperceptible when monitored solely from the xz-plane microscopic view, often creating a false impression of a symmetric bubble. This hidden nature of the deformation perfectly mirrors our aforementioned FEM simulation results, which theoretically predicted the extreme sensitivity of the *y*-axis thermal gradient. Therefore, these experimental observations unequivocally demonstrate that the precise spatial manipulation of the capillary—with a particular emphasis on *y*-axis alignment—is absolutely indispensable for synthesizing high-quality, geometrically symmetric microbubble resonators.

**Figure 6:**
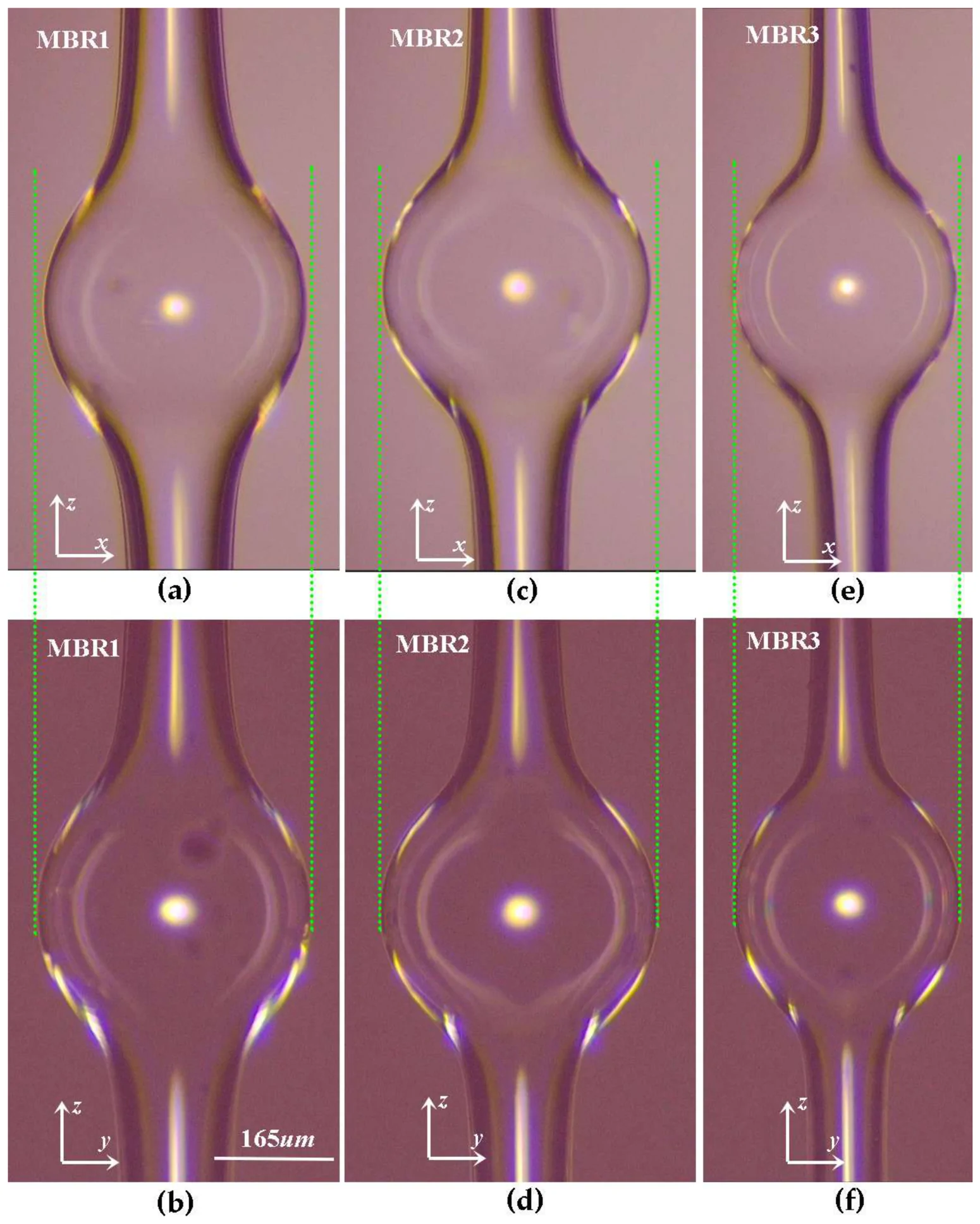
Microscopic images of the three fabricated MBRs. (**a**,**c**,**e**) present the xz-plane views, while (**b**,**d**,**f**) display the corresponding yz-plane views. Green dashed lines, extracted from the outer contours in the yz-plane, are superimposed to facilitate a direct comparison of the diametric expansion between the *y*- and *x*-axes. All images were captured at the exact same magnification.

Interestingly, the simulation reveals an intrinsic thermal anisotropy even when the capillary is perfectly positioned at the thermal center. As shown in Figure 4a, the temperature of the capillary wall along the *y*-axis is noticeably higher than that along the *x*-axis. Consequently, this thermal disparity inevitably induces a faster expansion rate in the transverse (*y*-axis) direction compared to the axial (*x*-axis) direction. This subtle anisotropic inflation is remarkably consistent with our experimental observations. Figure 6 presents the microscopic images of three successfully fabricated MBRs, captured from both the xz and yz-planes. Specifically, a segment of the capillary was pre-tapered over a hydrogen flame, and the three microbubbles were fabricated at regions with relatively thick, intermediate, and minimal (thinnest) capillary diameters, respectively. To facilitate a direct visual comparison, the outer contour of the microbubble in the yz-plane was traced with a white dashed line and superimposed onto the corresponding xz-plane image. Although the microbubbles exhibit excellent macroscopic symmetry in both directions, a meticulous comparison reveals that the diametric expansion in the yz-plane is indeed slightly larger than that in the xz-plane. This morphological discrepancy perfectly validates the temperature differential predicted by our simulation model. Based on our experimental experience, this inherent directional discrepancy can be effectively mitigated by incorporating a pre-tapering step. By locally heating and stretching the silica capillary over a hydrogen flame prior to the arc discharge, both the outer diameter and the wall thickness of the target segment are substantially reduced. Physically, this diminished cross-sectional area acts as a much smaller obstruction within the arc plasma, allowing the thermal energy to envelop the capillary more symmetrically. Driven by this homogenized thermal field, the resulting MBRs demonstrate a significantly improved three-dimensional spherical symmetry, as evidenced in Figure 6e,f. The fundamental electro-thermal mechanism governing this directional asymmetry and its geometric dependence will be thoroughly elucidated in the subsequent analysis.

To elucidate this seemingly counter-intuitive thermal distribution—where the transverse walls (*y*-axis) reach higher temperatures than the axial walls (*x*-axis) directly facing the electrodes—one must delve into the fundamental electro-thermal dynamics of the arc discharge. Unlike simple convective heat transfer, the thermal energy in this system is overwhelmingly dominated by the Joule heating of the plasma. Within this highly conductive plasma channel, the silica capillary functions as a perfect electrical insulator. Consequently, the electron flow propagating between the tungsten electrodes is strictly obstructed by the silica wall and is forced to divert and bypass the capillary.

This spatial diversion severely compresses the conductive path, inducing a pronounced “current crowding effect” [21,22] in the plasma regions immediately above and below the capillary (i.e., along the *y*-axis). According to the volumetric Joule heating equation, Q = J^2^/σ (where Q is the heating power density, J is the local current density, and σ is the electrical conductivity of the plasma), the drastically amplified current density at these transverse poles results in localized maximum heat generation. In stark contrast, the regions directly facing the electrodes along the *x*-axis act as an electrical “stagnation zone” or wake region, where the diverted current flow is minimal, leading to noticeably lower heat generation. This inherent electro-physical mechanism perfectly accounts for the simulated temperature disparity and fundamentally explains the preferential transverse expansion observed in our fabricated microbubbles.

### 4.2. Fabrication of In-Line Microbubble Arrays

Building upon the precise spatial and morphological control established in Section 4.1, our custom-built apparatus effectively overcomes the spatial limitation of conventional single-cavity fabrication. Unlike commercial optical fiber fusion splicers, which typically feature severely restricted axial moving ranges, our translation stage affords a significantly extended and highly precise travel distance along the capillary axis. This extended degree of freedom enables the sequential, step-by-step fabrication of multiple microcavities along a single, continuous silica capillary.

**Figure 7:**
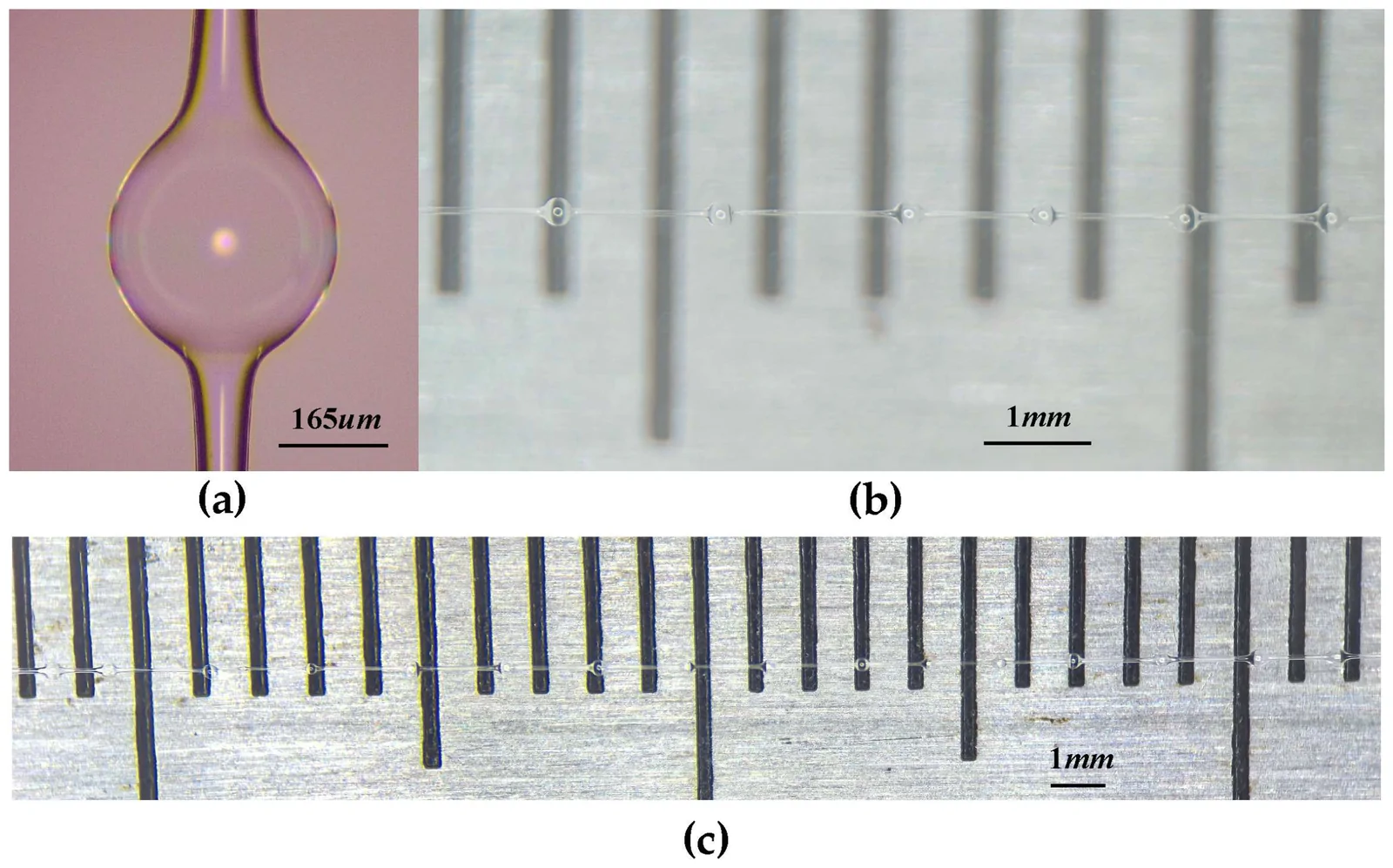
(**a**) Microscope image of a single microbubble resonator, (**b**) microscopic images of the fabricated in-line array of microbubbles, (**c**) panoramic view of 16 microbubble microcavities.

As depicted in Figure 7, an in-line array of 16 cascaded microbubble resonators was successfully synthesized on a single continuous capillary using our apparatus. Because the capillary underwent a hydrogen flame pre-tapering process, its diameter varies at different positions, which consequently leads to size variations among the fabricated microbubbles. Most importantly, despite these size variations across the array, our dynamic visual centering process successfully ensures that each individual microbubble achieves significantly improved three-dimensional spherical symmetry. Furthermore, as detailed in the subsequent optical characterization section, these cascaded cavities consistently retain exceptional optical quality, yielding ultra-high Q-factors. Because the series of microbubbles inherently shares a common, uninterrupted hollow microfluidic channel, it serves as an ideal physical platform for continuous fluid sample transport. Consequently, this in-line MBR array demonstrates tremendous potential for distributed microfluidic sensing and the development of highly integrated lab-on-a-chip (LOC) devices, where spatial and temporal variations in fluidic parameters can be simultaneously monitored across multiple sensing nodes.

### 4.3. Optical Characterization of the High-Q Resonator

**Figure 8:**
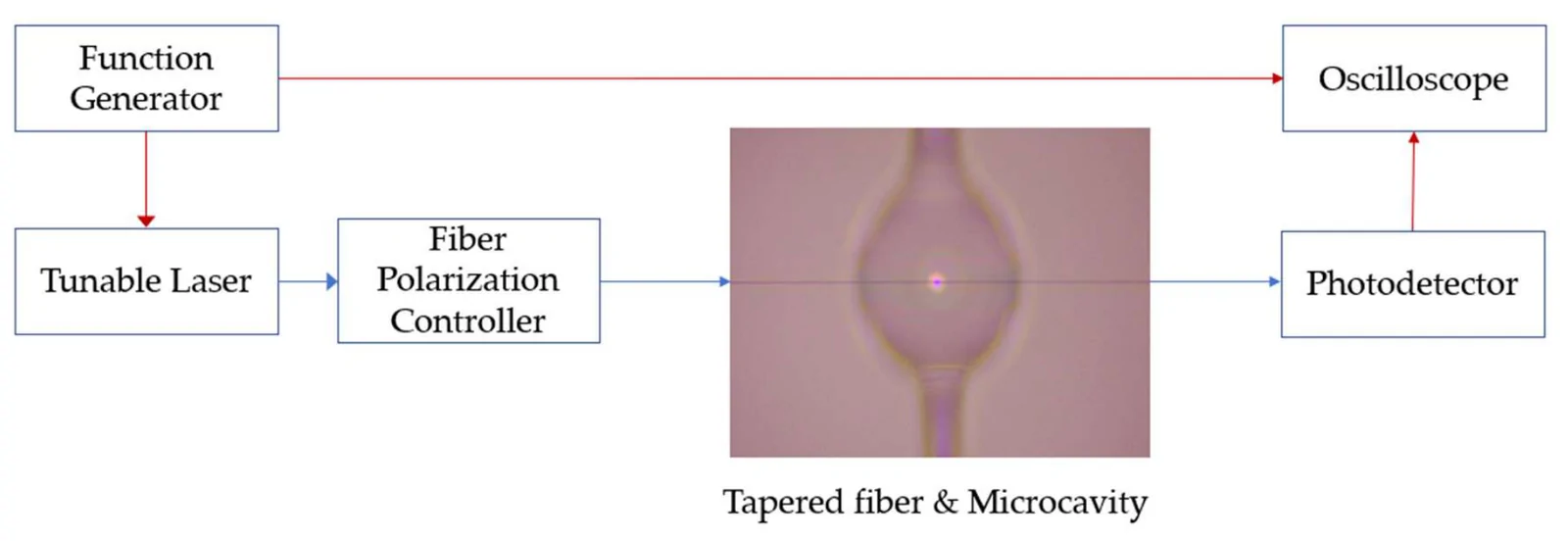
Schematic diagram of the WGM spectral measurement system. The inset shows the microscope image of coupling between the tapered fiber and the microbubble. Red arrows denote electrical-signal connections, and blue arrows denote optical-signal connections.

To quantitatively validate that the microbubbles fabricated by this optimized electro-thermal process preserve exceptional optical quality, the 16 microbubbles (shown in Figure 7) were subjected to whispering gallery mode (WGM) characterization. The transmission measurement setup is schematically illustrated in Figure 8. A narrow-linewidth, continuously tunable external-cavity diode laser (CTL 1500, TOPTICA Photonics, Graefelfing, Germany) was employed as the light source. The light was evanescently coupled into the equator of the selected microbubble via a tapered optical fiber. The tapered optical fiber was fabricated by heating with a hydrogen flame and drawing using an electronically controlled displacement stage, with a waist diameter of approximately 1–3 μm. The coupling between the tapered optical fiber and the microbubble was controlled by a piezoelectric displacement stage (MDT630, Thorlabs, Newton, MA, USA). The coupling distance was precisely controlled to ensure there was no physical contact between the fiber and the microbubble. A sub-wavelength air gap was maintained to ensure that the cavity operated in a slightly under-coupled regime. The transmitted optical signal was collected by a photodetector (PD) (PDA05CF2, Thorlabs, Newton, MA, USA) and subsequently recorded by a digital oscilloscope, which was synchronized with the trigger signal from the function generator.

**Figure 9:**
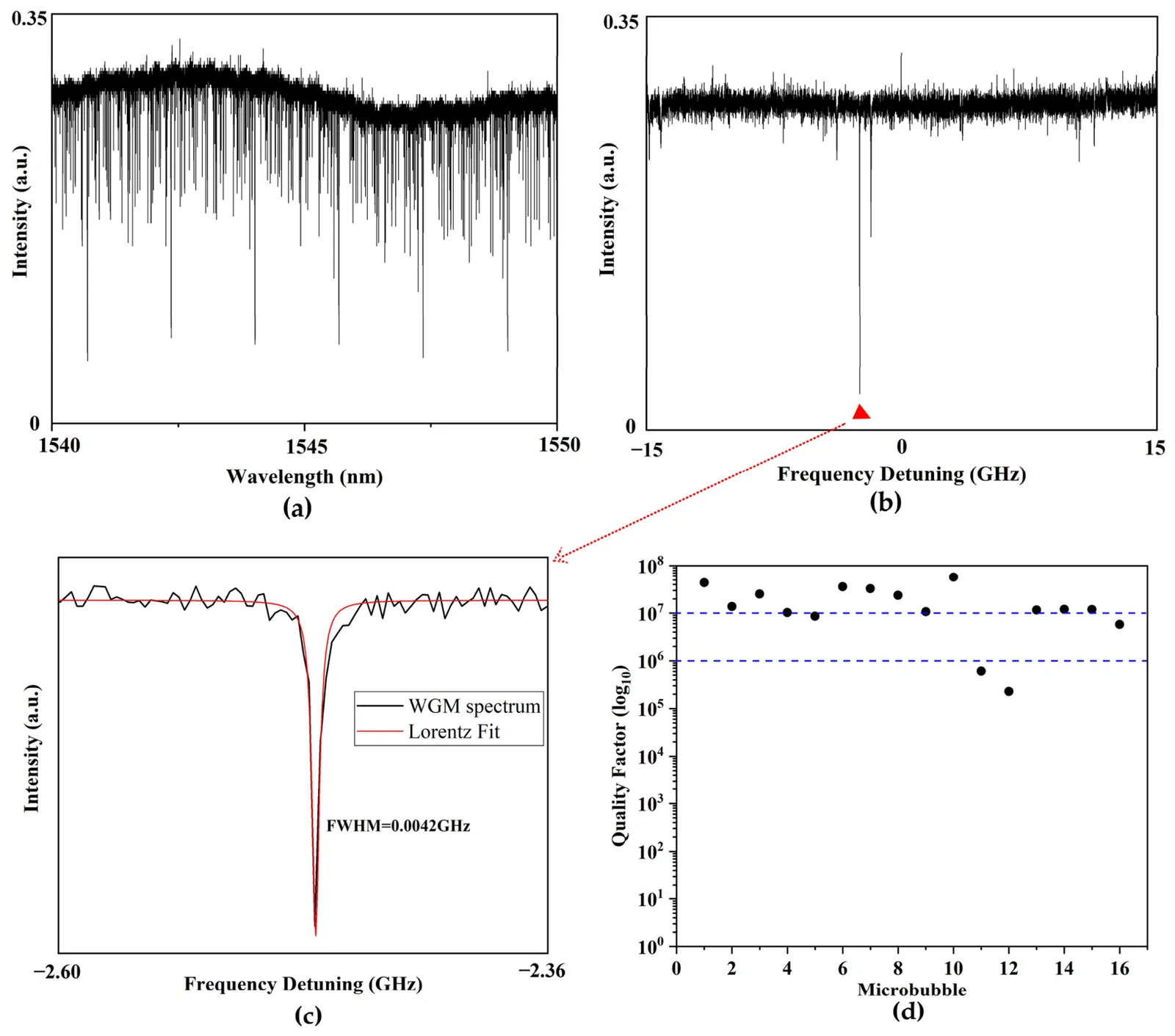
(**a**) Broad-range scanned WGM spectrum of the microbubble microcavity; (**b**) fine scanned WGM spectrum of the microcavity; (**c**) Lorentzian fitting performed on the dip indicated by the red triangle in panel (**b**); (**d**) Q-factor distribution of the 16 prepared microbubbles. The blue dashed lines mark the Q values of 10^7^ and 10^6^, respectively. Due to the logarithmic scale of the *y*-axis spanning three orders of magnitude, the error bars for the Q-factors are essentially smaller than the symbol size in the plot.

To comprehensively validate the reproducibility of our fabrication method and the optical performance of the in-line architecture, a newly synthesized array consisting of 16 cascaded MBRs was systematically characterized. Figure 9a displays the broadband transmission spectrum of a representative microcavity. The spectrum exhibits a dense cluster of sharp resonant dips, which is a typical hallmark of thin-walled microbubble resonators, indicating the successful excitation of high-order WGMs circulating within the cavity wall.

To accurately evaluate the intrinsic optical quality factor (Q-factor) while avoiding thermo-optic nonlinear effects, a fine scan was conducted by applying an external tuning signal to the laser. The fine scan spectrum is depicted in Figure 9b. A specific resonant mode—indicated by the red triangle in Figure 9b—was extracted for meticulous Lorentz fitting. As illustrated in Figure 9c, the experimental data of this narrow resonance dip match the standard Lorentzian line-shape function perfectly. The fitting results reveal an ultra-narrow full width at half maximum (FWHM, Δν) of merely 0.0042 GHz. By utilizing the fundamental relation Q = ν/Δν (where ν is the resonance frequency), the intrinsic Q-factor of this specific mode is calculated to be an impressive 4.62 × 10^7^.

The Q-factors for all 16 cascaded MBRs were systematically measured and calculated. The statistical results reveal a remarkably high yield: 12 out of the 16 microcavities successfully achieved ultra-high Q-factors exceeding the 10^7^ threshold. Additionally, two cavities demonstrated excellent Q-factors in the 10^6^ regime, while the remaining two yielded Q-factors on the order of 10^5^. Microscopic inspection indicated that the relatively lower Q-factors (~10^5^) in these two specific cavities were due to trace amounts of uncleaned polyimide coating residue left on the capillary surface at those specific nodes. Excluding these anomalies, the overall statistical data robustly confirm that our custom-built apparatus is highly capable of continuously and reliably fabricating in-line microbubble arrays with exceptional optical performance.

These characterization results unequivocally validate the superiority and reliability of our custom-built fabrication apparatus. It demonstrates that the proposed vision-assisted arc discharge method can successfully fabricate cascaded microbubble arrays while ensuring world-class optical Q-factors for individual cavities. Such outstanding optical confinement capability fully satisfies the stringent requirements for ultra-sensitive biochemical detection and advanced nonlinear optofluidic applications.

## 5. Conclusions

In conclusion, we have developed a highly controllable and cost-effective arc discharge system for the precise fabrication of high Q microbubble resonators and in-line microcavity arrays. To thoroughly elucidate the dynamics of the micro-glassblowing process, a finite-element electro-thermal model was established. The model reveals that the geometric symmetry of the MBR is fundamentally governed by its spatial alignment within the plasma arc. Specifically, the simulation and corresponding experimental results highlight a strict sensitivity to transverse (*y*-axis) misalignment, where even minute deviations lead to asymmetric inflation. Moreover, we discovered an intrinsic anisotropic heating phenomenon caused by the current crowding effect around the insulating silica capillary, which elegantly explains the subtle geometric discrepancies between the transverse and axial expansion rates. Relying on these theoretical insights, we utilized precision three-axis manipulation and in situ visual feedback to dynamically lock the optimal thermal center and mitigate asymmetric deformations. Consequently, we successfully broke through the spatial limitations of commercial fusion splicers, realizing continuous, in-line MBR arrays on a single capillary while ensuring ultra-high optical quality for individual cavities. Systematic optical characterization of a 16-element array demonstrated a remarkable yield, with 12 individual microcavities achieving ultra-high Q-factors exceeding 10^7^. This work not only provides a deep physical understanding of the arc-induced thermal shaping process but also delivers a robust technological platform for developing next-generation distributed optofluidic sensors and highly integrated lab-on-a-chip networks.

While the successful fabrication of these high-Q in-line MBR arrays lays a solid physical foundation for distributed microfluidic sensing and multiplexed detection, several critical experimental challenges remain to be addressed in future work to validate their practical applicability. First, developing robust, strain-free microfluidic packaging is imperative to protect the fragile thin walls during fluidic manipulation without degrading the intrinsic Q-factor. Second, achieving simultaneous evanescent coupling across multiple cascaded cavities necessitates the development of highly stable multi-tapered fiber arrays or customized prism-coupling platforms. Finally, real-time tracking of multiplexed resonance shifts requires advanced interrogation architectures to decouple adjacent sensing signals. Overcoming these engineering hurdles will be the focus of our ongoing research, ultimately translating these bare resonator arrays into fully functional, highly integrated lab-on-a-chip devices.

## Figures and Tables

**Table 1:** Benchmarking of the custom-built arc discharge system against state-of-the-art fabrication methods for microbubble resonators.

Parameters	Our Custom-Built Arc System	Commercial Fusion Splicers/Glass Processors	CO_2_ Laser Heating
Equipment Cost	Ultra-low (~$3000 USD)	High to Very High ($7000–$50,000+)	Very High ($20,000+)
Spatial Degree of Freedom	High (Independent 3-axis tuning for both electrodes and capillary)	Low (Fixed electrodes, restricted *Z*-axis stroke)	High (Requires complex beam alignment setup)
Discharge/Power Tunability	High (Wide-range voltage & time modulation)	Low (Highly automated/“black-box” programs)	High (Continuous power tuning)
Setup Footprint	Compact (Table-top)	Ultra-compact	Large (Requires an optical table)
In-Line Array Capability	Yes (Extended axial translation stage)	No/Highly restricted	Yes (Difficult to maintain focus over long spans)
Fabrication Time per Bubble	~5 to 8 min (Manual)	~2 to 5 min (Automated)	~2 to 5 min
Typical Q-factor Yield	>10^7^	~10^7^ (Limited by equipment obsolescence)	>10^7^

## Data Availability

The original contributions presented in this study are included in the article/Supplementary Material. Further inquiries can be directed to the corresponding author.

## Supplementary Materials

The following supporting information can be downloaded at: https://www.mdpi.com/article/10.3390/mi17091091/s1, Figures S1–S4: Microscopic images of a series of microbubbles fabricated by us. The background is a steel ruler with a minimum scale of 1 mm. Figures S5–S20: WGM spectra of 16 individual microbubbles, Lorentzian fits to the peak positions, and Q values calculated from the fitting results.
